# First record of the family Issidae (Hemiptera, Auchenorrhyncha, Fulgoroidea) from the Hawaiian Islands

**DOI:** 10.3897/BDJ.10.e80135

**Published:** 2022-03-22

**Authors:** Vladimir Gnezdilov, Charles R. Bartlett

**Affiliations:** 1 Zoological Institut RAS, St-Petersburg, Russia Zoological Institut RAS St-Petersburg Russia; 2 University of Delaware, Newark, United States of America University of Delaware Newark United States of America

**Keywords:** Issidae, new record, Pacific Region, Sarimini, Hawaii

## Abstract

**Background:**

*Euroxenusvayssieresi* (Bonfils, Attie & Reynaud, 2001) (Issinae, Sarimini) was described (in the genus *Borbonissus* Bonfils, Attie & Reynaud, 2001) from Réunion Island, in the Indian Ocean and, previous to this report, has not been recorded elsewhere. *Euroxenusvayssieresi* is here illustrated and re-described to improved taxonomic diagnosis.

**New information:**

*Euroxenusvayssieresi* is recorded for the first time from the Island of Hawaii in the Hawaiian Archipelago. This is first record of the family Issidae from the Hawaiian Archipelago.

## Introduction

The Hawaiian planthopper (Hemiptera, Auchenorrhyncha, Fulgoroidea) fauna consists of 207 endemic species (64 Cixiidae plus 143 Delphacidae) and at least 15 adventive species (10 Delphacidae, 2 Flatidae, 1 Tropiduchidae and 2 Derbidae) (Zimmerman 1948, Beardsley 1979, Beardsley 1990, Asche 1997, Asche 2000a, Asche 2000b, Nishida 2002, Hoch 2006). No additional adventive planthopper species have been reported since the publication of the Fourth Edition of Bishop Museum’s Hawaiian Terrestrial Arthropod Checklist (Nishida 2002), i.e. Matsunaga et al. (2019). Beardsley (1979) reported that the rate of 'accidental immigration and establishment' was about 16 species a year between 1937 and 1961 and about 19 species a year between 1962 and 1976 (subsequent rates have not been reported). The importation of live plant material may be the main source of immigrant species (at least for phytophagous insects) (Beardsley 1979, DeNitto et al. 2015, Mound et al. 2017) and the primary sources of introductions are the mainland, the United States and Asia-Pacific (DeNitto et al. 2015). Here, we report an established species of Issidae, found at three localities on the western side of the Island of Hawaii in 2021.

The planthopper family Issidae comprises about 1,090 species in 217 genera distributed worldwide (Bourgoin 2021). Issids are absent in southern Africa (except for two species of the genus *Ikonza* Hesse, 1925 from north Namibia and south Angola), Madagascar, the Seychelles and Tasmania (Gnezdilov 2013, Gnezdilov 2016). In Oceania, Issidae are known from New Guinea (eight genera, 17 species), the Solomon Islands (one species) and Fiji (one species) (Gnezdilov 2013, Gnezdilov et al. 2015, Gnezdilov 2020b), but are absent in Micronesia and Polynesia including New Zealand and Hawaii. In the South Indian Ocean, the Issidae are known only from the Mascarene Archipelago (two species, Bonfils et al. 2001), consisting (in part) of the large islands Mauritius, Réunion and Rodrigues.

The genus *Borbonissus* Bonfils, Attie & Reynaud, 2001 was described with two species from Réunion Island, *B.brunnifrons* Bonfils, Attie & Reynaud, 2001 (type species) and *B.vayssieresi* Bonfils, Attie & Reynaud, 2001 (Bonfils et al. 2001). Later, *Borbonissus* was synonymised under *Thabena* Stål 1866 and *Euroxenus* Gnezdilov, 2009, erected to accommodate *B.vayssieresi* (Gnezdilov 2009). Gnezdilov (2009) postulated that the issid fauna of Réunion Island had an Oriental genesis and proposed a close relationship of *Euroxenus* and the Oriental genus *Eusarima* Yang, 1994 (in Chan and Yang 1994), recently supported by molecular data (Gnezdilov et al. in press). *Thabenabrunnifrons* was later recorded from Rodrigues, Taiwan, Dongsha (Pratas) Islands and Singapore (Chan et al. 2013, Gnezdilov 2014b, Gnezdilov 2015).

Here, we record *E.vayssieresi* from the Island of Hawaii in the Hawaiian Archipelago. This species is re-described to improve on diagnostic features reported by Gnezdilov (2009) and Gnezdilov (2020).

## Materials and methods

Morphological terminology follows Anufriev and Emeljanov (1988) and Gnezdilov et al. (2014). Photographs were taken using a Canon EOS 5D Mark IV camera with the lens Canon-MP-E-65mm f/2,8 1-5x Macro and the flash Canon Macro Twin-Lite MT-26EX-RT. Images were produced using Helicon Focus v. 7.6.4 and Adobe Photoshop СС 2019 software. The genital segments of male specimens examined were macerated in 10% potassium hydroxide (KOH) and figured in glycerine jelly (Brunel Micro Ltd, UK) using a Leica MZ9.5 stereomicroscope with a camera lucida. The map was prepared using Google Earth Pro (version 7.3) with map data sources as attributed in image.

The specimens examined are deposited in University of Delaware, Department of Entomology and Wildlife Ecology, Newark, Delaware, USA (**UDCC**); Smithsonian Institution, National Museum of Natural History, Washington, DC (**USNM**); Bernice P Bishop Museum of Natural History, Honolulu, Hawaii (**BPBM**); and the Zoological Institute of the Russian Academy of Sciences, Saint Petersburg, Russian Federation (**ZIN**).

## Taxon treatments

### Euroxenus vayssieresi

(Bonfils, Attié & Reynaud, 2001)

B9495508-2525-5335-8A28-22AA690E8F63

https://www.gbif.org/species/2050778

= *Borbonissusvayssieresi* Bonfils, Attie & Reynaud, 2001: 220 (original description).
= *Euroxenusvayssieresi* (Bonfils, Attie & Reynaud, 2001), combination by Gnezdilov 2009: 84.
*Type locality.* Réunion, la Possession, Chemin des Anglais.

#### Materials

- **Type status:** Other material. **Occurrence:** catalogNumber: UDCC_TCN 00097423; recordedBy: W.D. Perreira; individualCount: 3; sex: Male; lifeStage: adult; **Taxon:** scientificName: *Euroxenusvayssieresi*; **Location:** country: USA; stateProvince: Hawaii; county: Hawaii; locality: Spencer Beach Park; verbatimElevation: 12 m; decimalLatitude: 20.02278; decimalLongitude: -155.82111; georeferenceProtocol: label; **Identification:** identifiedBy: V.M. Gnezdilov; dateIdentified: 2021; **Event:** samplingProtocol: Sweep Netting; eventDate: 13-08-2021; **Record Level:** ownerInstitutionCode: UDCC; basisOfRecord: PreservedSpecimen
- **Type status:** Other material. **Occurrence:** catalogNumber: UDCC_TCN 00097423; recordedBy: W.D. Perreira; individualCount: 3; sex: Female; lifeStage: adult; **Taxon:** scientificName: *Euroxenusvayssieresi*; **Location:** country: USA; stateProvince: Hawaii; county: Hawaii; locality: Spencer Beach Park; verbatimElevation: 12 m; decimalLatitude: 20.02278; decimalLongitude: -155.82111; georeferenceProtocol: label; **Identification:** identifiedBy: V.M. Gnezdilov; dateIdentified: 2021; **Event:** samplingProtocol: Sweep Netting; eventDate: 13-08-2021; **Record Level:** ownerInstitutionCode: UDCC; basisOfRecord: PreservedSpecimen
- **Type status:** Other material. **Occurrence:** catalogNumber: UDCC_TCN 00097423; recordedBy: W.D. Perreira; individualCount: 1; lifeStage: nymph; **Taxon:** scientificName: *Euroxenusvayssieresi*; **Location:** country: USA; stateProvince: Hawaii; county: Hawaii; locality: Spencer Beach Park; verbatimElevation: 12 m; decimalLatitude: 20.02278; decimalLongitude: -155.82111; georeferenceProtocol: label; **Identification:** identifiedBy: V.M. Gnezdilov; dateIdentified: 2021; **Event:** samplingProtocol: Sweep Netting; eventDate: 13-08-2021; **Record Level:** ownerInstitutionCode: UDCC; basisOfRecord: PreservedSpecimen
- **Type status:** Other material. **Occurrence:** catalogNumber: UDCC_TCN 00101252; recordedBy: W.D. Perreira; individualCount: 5; sex: Male; lifeStage: adult; **Taxon:** scientificName: *Euroxenusvayssieresi*; **Location:** country: USA; stateProvince: Hawaii; county: Hawaii; locality: Spencer Beach Park; verbatimElevation: 12 m; decimalLatitude: 20.02278; decimalLongitude: -155.82111; georeferenceProtocol: label; **Identification:** identifiedBy: V.M. Gnezdilov; dateIdentified: 2021; **Event:** samplingProtocol: Sweep Netting; eventDate: 16-07-2021; **Record Level:** ownerInstitutionCode: UDCC, ZIN; basisOfRecord: PreservedSpecimen
- **Type status:** Other material. **Occurrence:** catalogNumber: UDCC_TCN 00101401; recordedBy: W.D. Perreira; individualCount: 8; sex: Male; lifeStage: adult; **Taxon:** scientificName: *Euroxenusvayssieresi*; **Location:** country: USA; stateProvince: Hawaii; county: Hawaii; locality: Spencer Beach Park; verbatimElevation: 12 m; decimalLatitude: 20.02278; decimalLongitude: -155.82111; georeferenceProtocol: label; **Identification:** identifiedBy: V.M. Gnezdilov; dateIdentified: 2021; **Event:** samplingProtocol: Sweep Netting; eventDate: 03-07-2021; **Record Level:** ownerInstitutionCode: UDCC, USNM, BPBM; basisOfRecord: PreservedSpecimen
- **Type status:** Other material. **Occurrence:** catalogNumber: UDCC_TCN 00101405; recordedBy: W.D. Perreira; individualCount: 5; sex: Female; lifeStage: adult; **Taxon:** scientificName: *Euroxenusvayssieresi*; **Location:** country: USA; stateProvince: Hawaii; county: Hawaii; locality: Spencer Beach Park; verbatimElevation: 12 m; decimalLatitude: 20.02278; decimalLongitude: -155.82111; georeferenceProtocol: label; **Identification:** identifiedBy: V.M. Gnezdilov; dateIdentified: 2021; **Event:** samplingProtocol: Sweep Netting; eventDate: 03-07-2021; **Record Level:** ownerInstitutionCode: UDCC, ZIN; basisOfRecord: PreservedSpecimen
- **Type status:** Other material. **Occurrence:** catalogNumber: UDCC_TCN 00101414; recordedBy: W.D. Perreira; individualCount: 4; sex: Female; lifeStage: adult; **Taxon:** scientificName: *Euroxenusvayssieresi*; **Location:** country: USA; stateProvince: Hawaii; county: Hawaii; locality: Spencer Beach Park; verbatimElevation: 12 m; decimalLatitude: 20.02278; decimalLongitude: -155.82111; georeferenceProtocol: label; **Identification:** identifiedBy: V.M. Gnezdilov; dateIdentified: 2021; **Event:** samplingProtocol: Sweep Netting; eventDate: 16-07-2021; **Record Level:** ownerInstitutionCode: UDCC, ZIN; basisOfRecord: PreservedSpecimen
- **Type status:** Other material. **Occurrence:** catalogNumber: UDCC_TCN 00101417; recordedBy: W.D. Perreira, N.G. Miller, and D.A. Yee; individualCount: 1; sex: Male; lifeStage: adult; **Taxon:** scientificName: *Euroxenusvayssieresi*; **Location:** country: USA; stateProvince: Hawaii; county: Hawaii; locality: Keauhou Senic Lookout; verbatimElevation: 91 m; decimalLatitude: 19.57611; decimalLongitude: -155.95917; georeferenceProtocol: label; **Identification:** identifiedBy: V.M. Gnezdilov; dateIdentified: 2021; **Event:** samplingProtocol: Sticky Trap; eventDate: 10/14-05-2021; **Record Level:** ownerInstitutionCode: UDCC; basisOfRecord: PreservedSpecimen
- **Type status:** Other material. **Occurrence:** catalogNumber: UDCC_TCN 00101418; recordedBy: W.D. Perreira; individualCount: 1; sex: Female; lifeStage: adult; **Taxon:** scientificName: *Euroxenusvayssieresi*; **Location:** country: USA; stateProvince: Hawaii; county: Hawaii; locality: HonoKohau Harbor; verbatimElevation: 3 m; decimalLatitude: 19.67111; decimalLongitude: -156.02056; georeferenceProtocol: label; **Identification:** identifiedBy: V.M. Gnezdilov; dateIdentified: 2021; **Event:** samplingProtocol: Sticky Trap; eventDate: 02/16-07-2021; **Record Level:** ownerInstitutionCode: UDCC; basisOfRecord: PreservedSpecimen
- **Type status:** Other material. **Occurrence:** catalogNumber: UDCC_TCN 00101436; recordedBy: W.D. Perreira; individualCount: 1; sex: Male; lifeStage: adult; **Taxon:** scientificName: *Euroxenusvayssieresi*; **Location:** country: USA; stateProvince: Hawaii; county: Hawaii; locality: Spencer Beach Park; verbatimElevation: 12 m; decimalLatitude: 20.02278; decimalLongitude: -155.82111; georeferenceProtocol: label; **Identification:** identifiedBy: V.M. Gnezdilov; dateIdentified: 2021; **Event:** samplingProtocol: Sweep Netting; eventDate: 03-07-2021; **Record Level:** ownerInstitutionCode: UDCC; basisOfRecord: PreservedSpecimen
- **Type status:** Other material. **Occurrence:** catalogNumber: UDCC_TCN 00101437; recordedBy: W.D. Perreira; individualCount: 1; sex: Female; lifeStage: adult; **Taxon:** scientificName: *Euroxenusvayssieresi*; **Location:** country: USA; stateProvince: Hawaii; county: Hawaii; locality: Spencer Beach Park; verbatimElevation: 12 m; decimalLatitude: 20.02278; decimalLongitude: -155.82111; georeferenceProtocol: label; **Identification:** identifiedBy: V.M. Gnezdilov; dateIdentified: 2021; **Event:** samplingProtocol: Sweep Netting; eventDate: 03-07-2021; **Record Level:** ownerInstitutionCode: UDCC; basisOfRecord: PreservedSpecimen

#### Description

**Figure 1.**
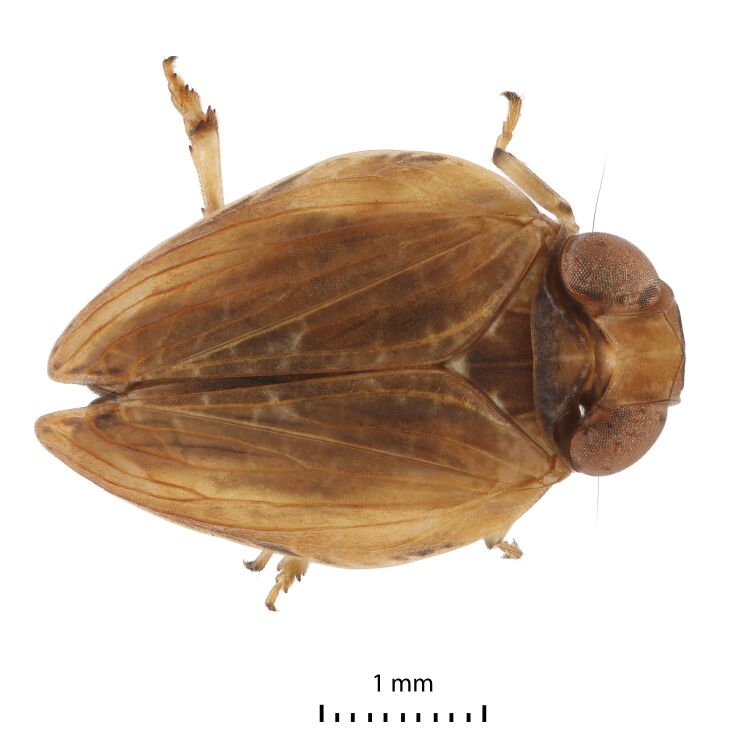
*Euroxenusvayssieresi*, Hawaii Island, male dorsal view.

**Figure 2.**
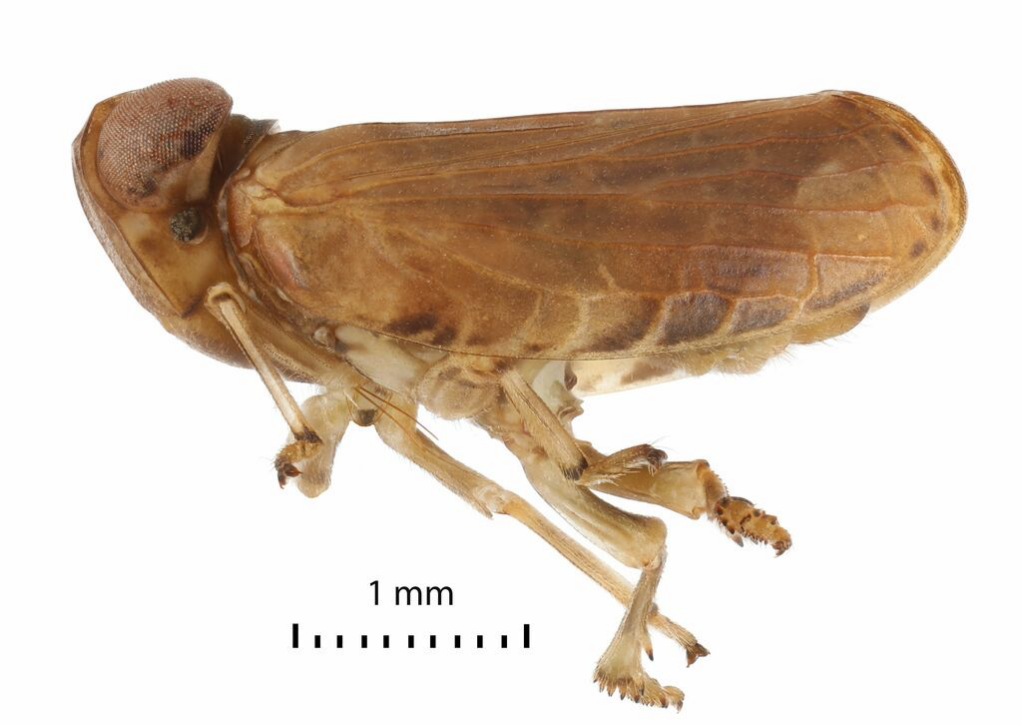
*Euroxenusvayssieresi*, Hawaii Island, male left lateral view.

**Figure 3.**
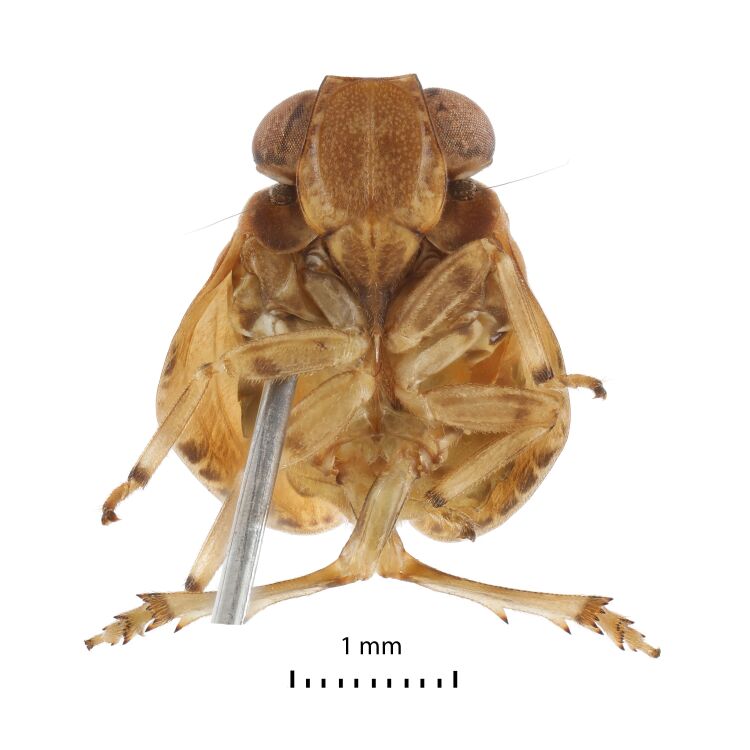
*Euroxenusvayssieresi*, Hawaii Island, male frontal view.

**Figure 4.**
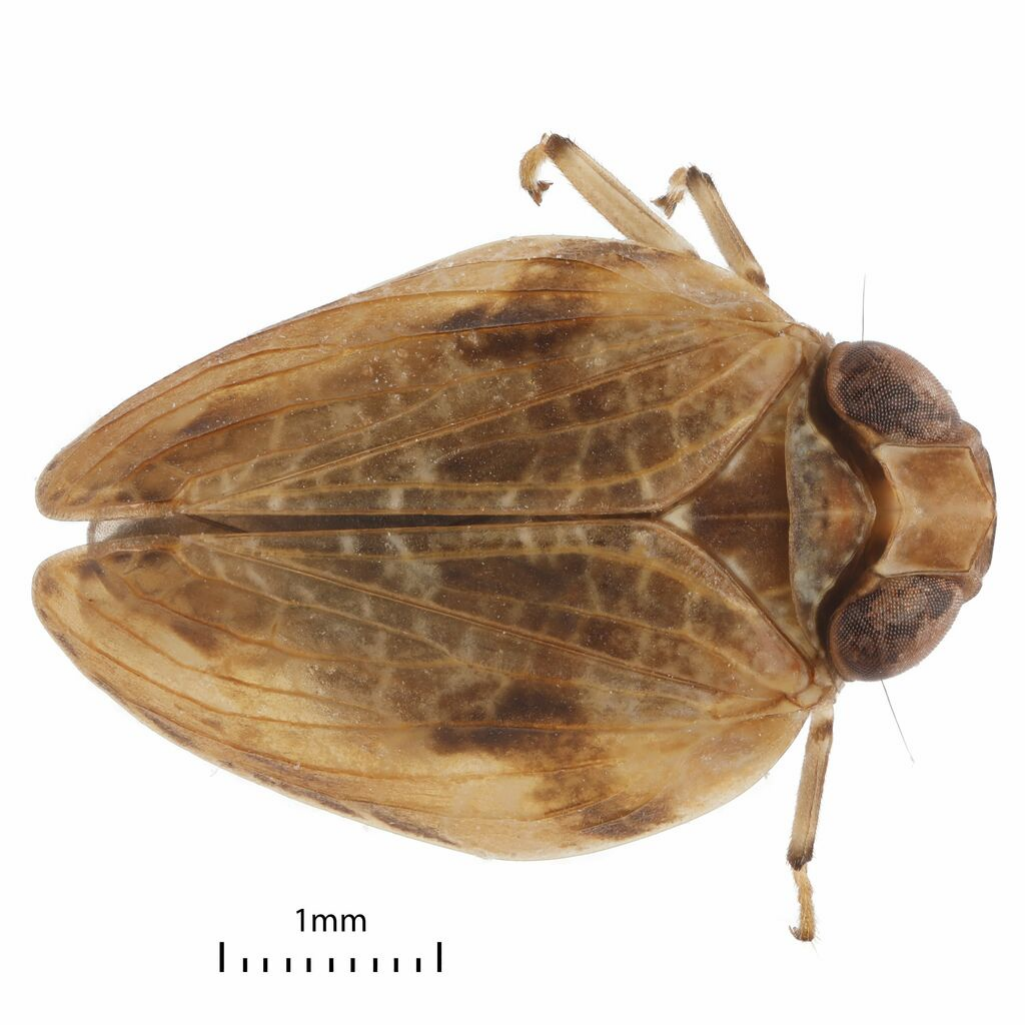
*Euroxenusvayssieresi*, Hawaii Island, female dorsal view.

Colouration. General colouration light brown-yellowish to dark brown on anteclypeus and forewing clavus (Figs 1, 2, 3, 4). Pedicel, apices of fore and middle tibiae and apices of spines black. Forewings with black marginal cells along the costal and lateral margins (Figs 1, 2) and sometimes (Fig. 4) with dark brown band from the costal margin to inner claval margin in basal half on females. Hind femora and outer surfaces of hind tibiae dark brown. Hind margins of gonostyli dark brown to black on dorsolateral angles. Ungues dark brown to black. Gonoplacs dark brown.

Structure. Body length, males and females – 4.0 mm. Metope (~ frons, Fig. 3) broad, widest below the eyes, with distinct median carina running from upper margin through postclypeus; sublateral carinae distinct, joined with median carina at metopial upper margin (carinae somewhat projecting along upper margin), almost reaching metopoclypeal suture. Postclypeus broadly rhomboid. Rostrum reaching hind coxae, 2^nd^ and 3^rd^ segments are almost equal in length; 3^rd^ one narrowing apically. Antennal scape very short, pedicel globular, just longer than wide. Coryphe (~ vertex, Figs 1, 4) as concave hexagon, slightly wider than long medially, anterior margin produced, obtusely angled, posterior margin concave. Metope and coryphe (in lateral view, Fig. 2) joined at obtuse angle (dorsal portion of sublateral carinae somewhat projecting). Ocelli vestigial. Eyes large, each eye nearly as wide as coryphe.

**Figure 5.**
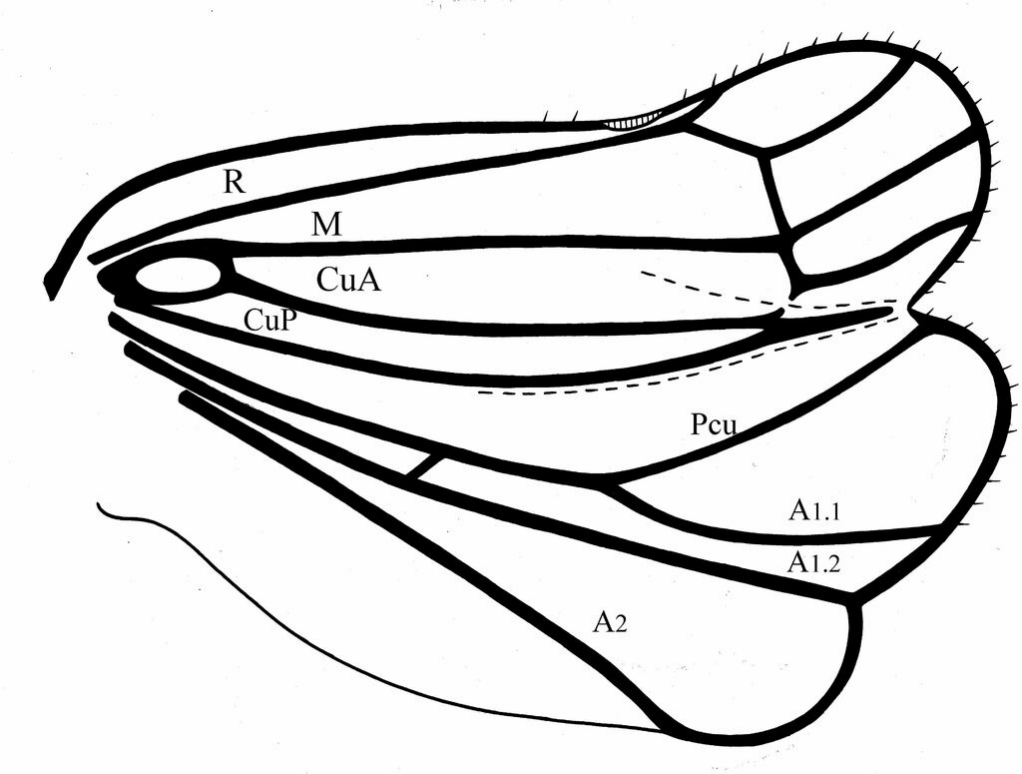
*Euroxenusvayssieresi* hindwing venation. Abbreviations: R = radius, M = media, CuA = anterior cubitus, CuP = posterior cubitus, Pcu = postcubitus, A1.1 = anterior branch of first anal vein; A1.2 = posterior branch of first anal vein; A2 = second anal vein.

Pronotum slightly shorter than coryphe (Figs 1, 4), with smooth median carina, anterior margin strongly convex, posterior margin nearly straight, with weak median concavity. Paradiscal fields very narrow behind eyes, paranotal lobes flat, apically rounded. Mesonotum length 1.5 times pronotum at mid-line, with median and lateral carinae. Tegulae large. Forewing slightly narrowing apically (Bonfils et al. 2001, fig. 22) (Figs 1, 2, 4), without hypocostal plate and with wide precostal area; clavus long, 4/5 of wing length, broadly triangular. Forewing vein sequence: R 2, furcating near basal cell; M 2, furcating after wing middle; CuA 2, furcating after wing middle; basal cell narrowly oval. Hind wing with coupling lobe and deep cubital and vannal clefts (Fig. 5); anal lobe less wide than remigial and remigio-vannal lobes. Hind wing vein sequence: R 2, furcating apically after coupling lobe; r-m 1; M 1; m-cua 1; CuA 2; CuP 1; Pcu 1; A_1_ 2; A_2_ 1. CuA_2_ and CuP fused and flattened apically and Pcu and A_1.1_ fused medially. Front and middle femora slightly flattened. Hind tibia with two large lateral spines past mid-length and six apical spines. First and second metataromeres of subequal length, but basitarsus wider; basitarsus with two latero-apical spines and 13 intermediate spines arranged in arc; ventral surface with long setae; second metatarsomere with pair of latero-apical spines and median lobe.

**Figure 6.**
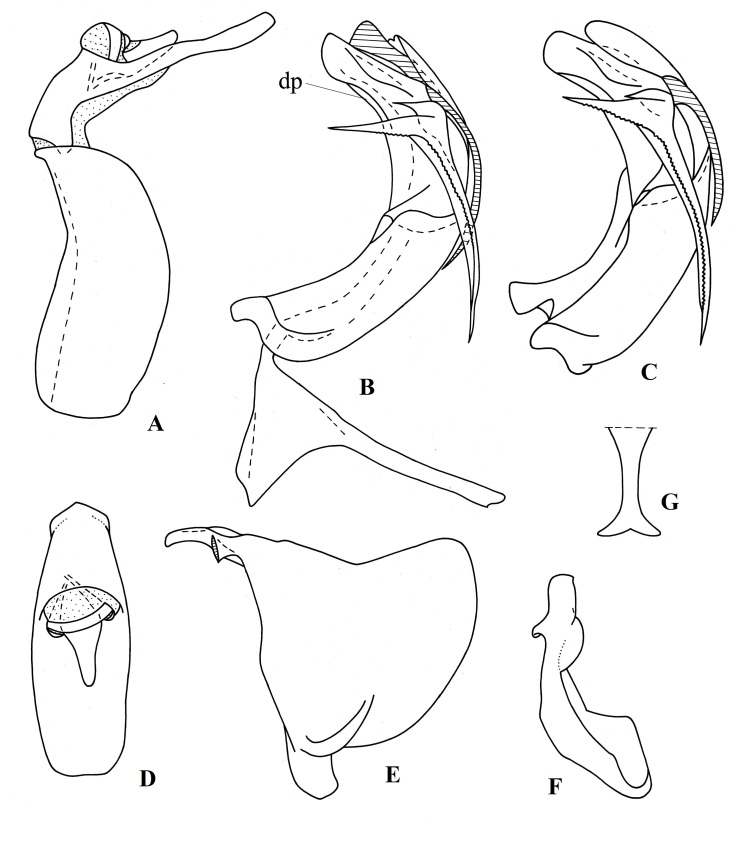
*Euroxenusvayssieresi*, male terminalia (A–B, D-G – Hawaii; C – Réunion Island, La Possession) **A** pygofer and anal tube, lateral view; **B** penis and connective (apical aedeagal processes shaded), lateral view; **C** penis (apical aedeagal processes not visible), lateral view; **D** anal tube, dorsal view; **E** gonostylus, lateral view; **F** gonostylus, dorsal view; **G** connective handle, dorso-caudal view. Abbreviation: dp – dorsal process of the phallobase.

Male terminalia: Pygofer (in lateral view, Fig. 6A) elongate vertically, with convex hind margins. Hind margin of sternite VII straight (ventral view). Phallobase (Fig. 6B and C) narrow and strongly curved (in lateral view), with a narrow process dorsally (Fig. 6B *dp*); each dorsolateral phallobase lobe with three-branched large process – two branches directed dorsally and the longest branch directed basally, with serrulate carina. Each dorsolateral phallobase lobe (in lateral view) with a lateral slit and folds near middle and with narrow semicircular subapical lobe. Ventral phallobase lobe long and wide, with an apical notch. Aedeagus with a pair of long (0.3× length of phallobase, Fig. 6B and C), narrow, apically pointed ventral hooks, arising subapically and directed basally. Apical aedeagal processes long and wide apically (Fig. 6B, shaded). Gonostylus (Fig. 6E) with deeply concave hind margin. Capitulum wide, not narrowing apically (in dorsal view, Fig. 6F), with small lateral tooth (in lateral view). Connective (lateral view, Fig. 6B) with small cup and long handle, in caudal view (Fig. 6G), with ventral margin bilobed (these articulating with the gonostyli). Anal tube elongate (Fig. 6A), in dorsal view (Fig. 6D) nearly 3 times as long as wide medially, slightly narrowing apically, with weak apical concavity. Anal column 0.3x as long as anal tube, narrow.

Female terminalia: Hind margin of sternite VII protruding medially (ventral view). Gonoplacs short. Anal tube elongate. Anal column short, 0.25 of anal tube length, narrow.

Notes: *Gnezdilov (2020)* (in fig. 2) had not observed a small basal process of three-branched processes of dorsolateral phallobase lobes (Fig. 6B and C) on his drawings of male genitalia of *E.vayssieresi* from Réunion Island (Saint-Paul); otherwise no significant differences in external morphological features were found between the specimens from Réunion (La Possession and Saint-Paul) and those of the Hawaii Islands.

#### *Euroxenus* Gnezdilov, 2009

Genus diagnosis: Metope (~ frons) broad (Fig. 3), widest below the eyes, with distinct median carina running from upper margin through postclypeus and distinct sublateral carinae joined with median carina at upper metopial margin and almost reaching metopoclypeal suture. Rostrum reaching hind coxae, 2^nd^ and 3^rd^ segments are almost equal in length; 3^rd^ narrowing apically. Coryphe (~ vertex) (Fig. 4) slightly wider than long medially, anterior margin produced, obtusely angled. Metope and coryphe joined at obtuse angle (in lateral view, Fig. 2). Paradiscal fields of pronotum very narrow behind eyes. Forewings just covering abdominal apex, slightly narrowing apically, without hypocostal plate, with wide precostal area; clavus long. Forewing vein sequence: R 2, furcating near basal cell; M 2, CuA 2, both furcating after wing middle. Hind wings (Fig. 5) with deep cubital and vannal clefts; anal lobe less wide than remigial and remigio-vannal lobes. Hind wing vein sequence: R 2; r-m 1; M 1; m-cua 1; CuA 2; CuP 1; Pcu 1; A_1_ 2; A_2_ 1. CuA and CuP fused and flattened apically and Pcu and A_1_ fused medially. Hind tibia with two large lateral spines. First metataromere with two latero-apical spines and 13 intermediate spines arranged in arc. Phallobase narrow, curved (in lateral view, Fig. 6B and C), each dorsolateral phallobase lobe with narrow semicircular subapical lobe, large three-branched process below it and with a lateral slit and folds near middle. Ventral phallobase lobe long and wide. Aedeagus with a pair of long and narrow, apically pointed ventral hooks, arising subapically and directed basally. Apical aedeagal processes long and wide apically. Gonostylus (in lateral view, Fig. 6E) with deeply concave hind margin. Hind margin of female sternite VII protruding medially (ventral view); gonoplacs short; anal tube elongate.

Differential genus diagnosis: *Euroxenus* is closely related to *Eusarima* Yang, 1994 and *Duplexissus* Wang, Zhang & Bourgoin, 2019, based on having completely developed median and sublateral carinae of metope and the presence of a long and narrow process on each dorso-lateral lobe of the phallobase. However, *Euroxenus* differs from both genera by the shorter forewings with bifurcate median veins (tri- or tetrafurcate in *Eusarima* and *Duplexissus*) and by trifurcate processes of the phallobase (simple in *Eusarima* and bifurcate in *Duplexissus*) (Chan and Yang 1994, figs. 45–72; Wang et al. 2019, figs. 12-22).

## Discussion

**Figure 7.**
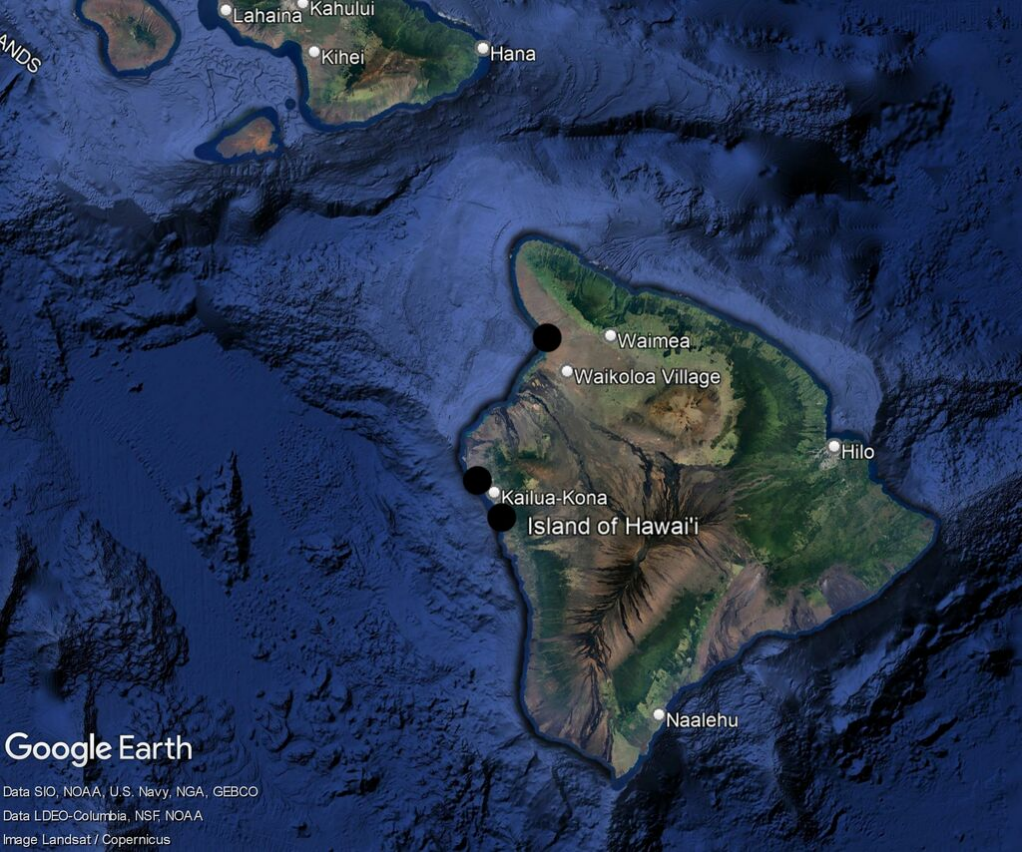
Collecting localities for *Euroxenusvayssieresi* on the Island of Hawaii (black circles). Map created using Google Earth, map data sources provided in the lower left of the image.

The specimens of *Euroxenusvayssieresi* were all collected in 2021 in very dry areas, on the west side of the Island of Hawaii at three localities (Fig. 7, from north to south): Keauhou, Kailua-Kona and Spencer Beach Park. The three localities are all near the coast (Keauhou Lookout is about ca. 300 feet (ca. 100 m) elevation), with the straight-line distance between Keauhou and Spencer Beach about 30 miles (48 km). The dominant vegetation at the sites was exotic species with a Kiawe (*Prosopispallida*) and Koa Haole (*Leucaenaleucocephala*) canopy. The specimens were mainly collected by general sweeping of grasses and weeds of the understorey, with no clear plant associations made.

In Réunion, adults and nymphs of *E.vayssieresi* were recorded from *Cestrum* sp. (stated as ‘*noctuiflorum*’, but apparently meaning *nocturnum*, Bourgoin 2021) and *Capsicum* sp. (Solanaceae), *Brideliamicrantha* (Phyllanthaceae) and *Coccolobauvifera* (Polygonaceae) suggesting that this species is polyphagous (Bonfils et al. 2001, Attié et al. 2008). Bonfils et al. (2001) noted that their specimens were collected in semi-dry secondary vegetation on exotic and native plants located on the northwest and west of the "leeward" region of Réunion, which is similar to the circumstances where this species was found in Hawaii.

The genus *Euroxenus* (Issinae, Sarimini sensu Wang et al. 2016 and Gnezdilov et al. 2020) was monotypic as described. Gnezdilov (2020) placed the monotypic genus *Duplexissus* Wang, Zhang & Bourgoin, 2019 (Wang et al. 2019) in synonymy under *Euroxenus*, but this placement was disputed by Wang et al. (2020) on both morphological and molecular grounds.

Issids are not typically good dispersers (many are sub-brachypterous and flightless) and transport of *Euroxenusvayssieresi* to Hawaii is most likely by human agency and in association with live plants. This was previously hypothesised as the mode of transport for other issid species (Gnezdilov and O'Brien 2006, Gnezdilov 2009).

## Supplementary Material

XML Treatment for Euroxenus vayssieresi
